# End-colostomy diverticulitis with parastomal phlegmon: A case report

**DOI:** 10.1097/MD.0000000000008358

**Published:** 2017-10-27

**Authors:** Mirza Muradbegovic, Pénélope St-Amour, David Martin, David Petermann, Samir Benabidallah, Luca Di Mare

**Affiliations:** a Department of General and Visceral Surgery, EHC Hospital, Morges; b Department of Visceral Surgery, University Hospital CHUV, Lausanne; c Unilabs, Department of Pathology, Lausanne, Switzerland.

**Keywords:** acute diverticulitis, end-colostomy, segmental colonic resection

## Abstract

**Rationale:**

Acute colonic diverticulitis is a well-known surgical emergency, which occurs in about 10 percent of patients known for diverticulosis.

**Patient concerns:**

The case of a 77-year-old woman is reported, with past history of abdominoperineal resection with end-colostomy for low rectal adenocarcinoma, and who developed an acute colonic diverticulitis in a subcutaneous portion of colostomy with parastomal phlegmon.

**Diagnoses:**

Initial computed tomography imaging demonstrated a significant submucosal parietal edema with local fat tissues infiltration in regard of 3 diverticula.

**Interventions:**

A two-step treatment was decided: first a nonoperative treatment was initiated with 2 weeks antibiotics administration, followed by, 6 weeks after, a segmental resection of the terminal portion of the colon with redo of a new colostomy by direct open approach.

**Outcomes:**

Patient was discharged on the second postoperative day without complications. Follow-up at 2 weeks revealed centimetric dehiscence of the stoma, which was managed conservatively until sixth postoperative week by stomatherapists.

**Lessons subsections:**

Treatment of acute diverticulitis with parastomal phlegmon in a patient with end-colostomy could primary be nonoperative. Delayed surgical treatment with segmental colonic resection was proposed to avoid recurrence and potential associated complications.

## 1 Introduction

Prevalence of colonic diverticulosis is age-dependent, increasing up to 60 percent at age of 60.^[1]^ The natural history of acute diverticulitis is mild and most patients are treated successfully by conservative measures.^[2]^ However, 4% to 15% of those patients will develop acute diverticulitis.^[1]^

The case of an acute colonic diverticulitis in a subcutaneous terminal portion of an end-colostomy is reported.

## 2 Case report

A 77-year-old woman, with medical history of abdominoperineal resection with end-colostomy for low sphincters-infiltrating rectal adenocarcinoma 8 years ago, presented a 3-day history of acute parastomal pain and erythema, without other associated symptoms. The pain radiated from lateral part of stoma to the left flank. Last colonoscopy had been performed 2 years ago, and had shown pancolic diverticulosis.

**Figure 1:**
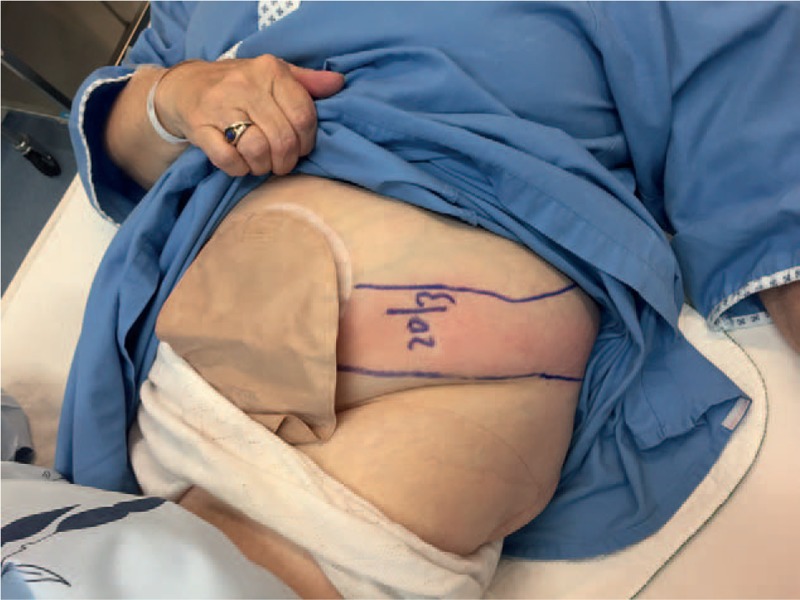
Clinical examination: erythema taking origin at the stoma and extending to left flank associated with tenderness.

On physical examination, an erythematous phlegmon taking origin from lateral part of stoma and propagating to the left flank was palpable, with severe tenderness (Fig. 1). Stoma digital examination indentified pain in left parietal part of end-colostomy on the first 4 cm. Otherwise, the rest of abdomen was soft with normal bowel sounds. Vital parameters were normal.

Blood sample analysis highlighted an inflammatory syndrome with an increased C-reactive protein (83 mg/L, normal range 0–10 mg/L) and normal leucocytes count (7.7 G/L, normal range 4.5–11.5 G/L).

**Figure 2:**
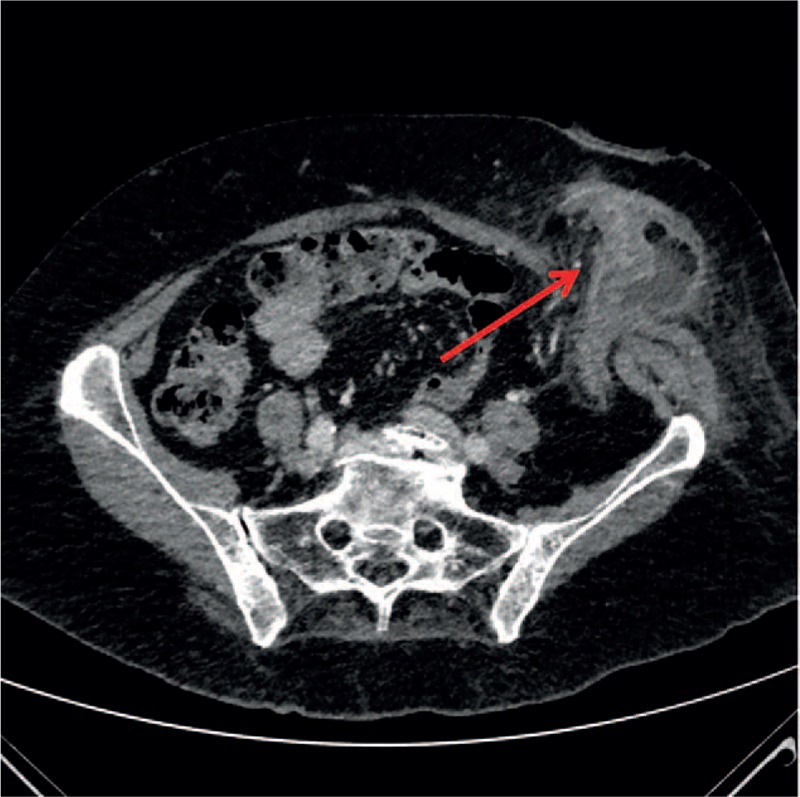
Computed tomography: acute noncomplicated diverticulitis of end-colostomy (red arrow).

Computed tomography imaging demonstrated parietal inflammation of the colonic handle of the stoma with bundle of fluid in regard of 3 diverticula, and therefore compatible with a noncomplicated diverticulitis (Fig. 2).

**Figure 3:**
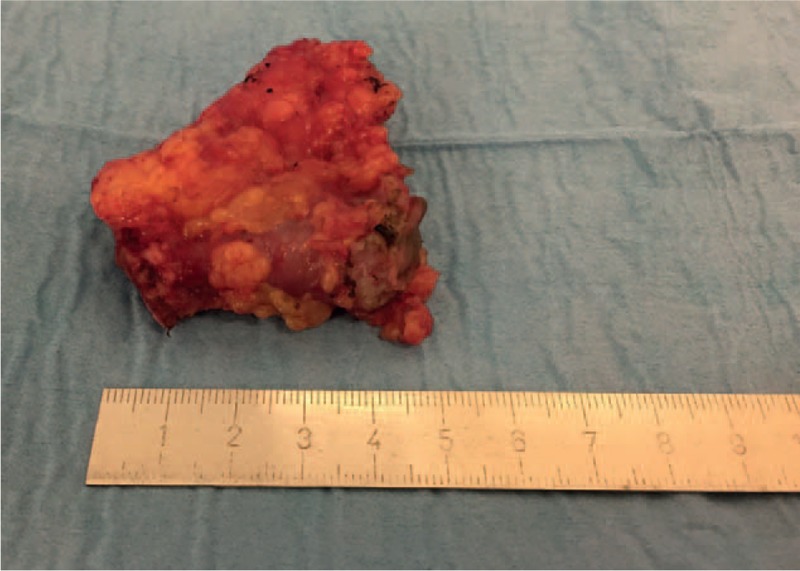
Fresh operative piece of the end-colostomy removed.

A 2-step treatment was initiated. During the initial hospitalization, an intravenous antibiotic treatment with ciprofloxacin (quinolone) and metronidazole (nitroimidazole) was initiated for 72 hours, with a relay per os for 10 days in total. The patient was discharged after 3 days in enhanced clinical and biological state. A new colonoscopy was performed 2 weeks after hospital discharge, confirming the pancolic diverticulosis. Eight weeks after the acute phase, elective surgical management was carried out through direct stoma approach to perform segmental colonic resection of the subcutaneous part of end-colostomy (Fig. 3). A new end-colostomy was made at the same site. Patient was discharged on postoperative day 2 without any complications.

**Figure 4:**
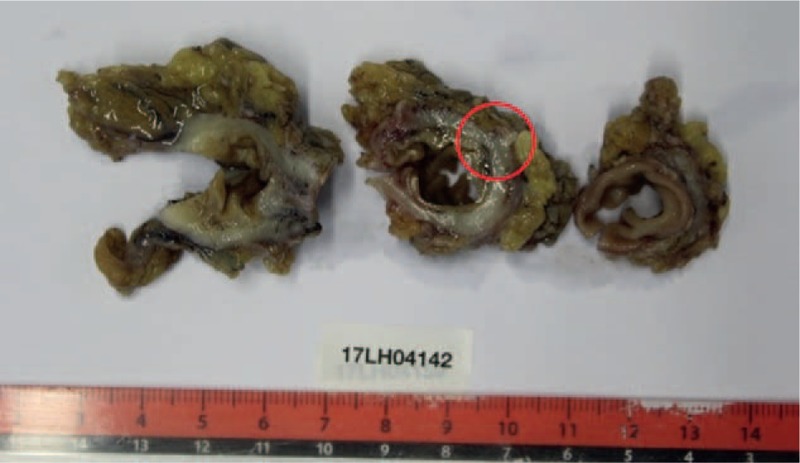
Surgical specimen sliced in 3 pieces with evidence of a diverticular lesion without sign of perforation (red circle).

**Figure 5:**
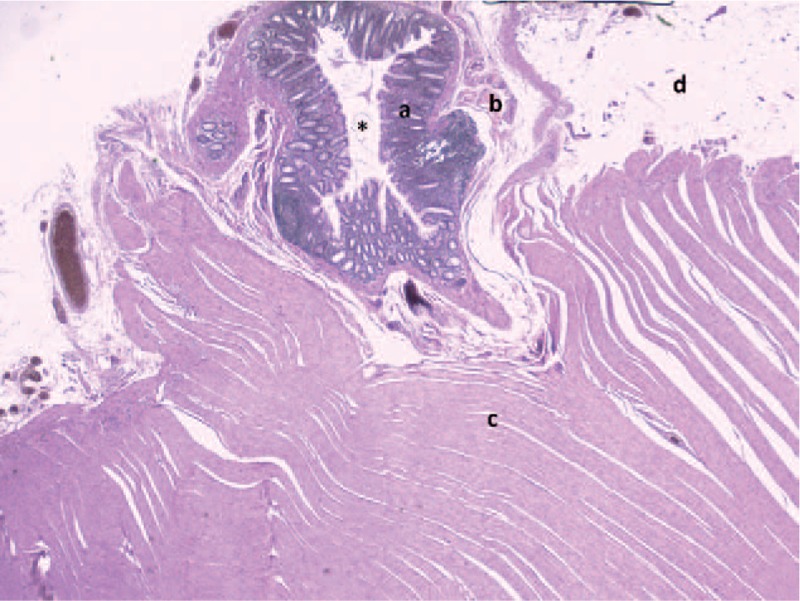
Microscopic view with hematoxylin and eosin coloration showed uncomplicated diverticular lesion. ∗ Lumen of the diverticulum, ^a^Mucosa of the diverticulum, ^b^Submucosa with muscularis mucosae of the diverticulum, ^c^Muscularis of the colon, ^d^ Serosa of the colon.

Histopathology confirmed the presence of diverticular disease (Figs. 4 and 5). Follow-up at 2 weeks showed an incision dehiscence of the stoma, managed by stomatherapists during 4 more weeks.

## 3 Discussion

Diverticular disease of the colon constitutes important reason for emergency consultation, hospital admission and a significant part of healthcare costs in Western countries.^[3]^ From those patients with diverticulosis, around 4 to 15 percent will develop an acute diverticulitis.^[4–6]^ The mean age of onset of diverticulitis is 63 years.^[7]^

This current case of acute diverticulitis with additional parastomal phlegmon developed a few years after stoma creation, as described in another case of the literature, 16 years after initial surgery.^[8]^ Residual diverticula in the remaining colon were diagnosed previously in both patients. In our case, clinical findings were more typical for parastomal hernia and associated complications, as perforation and abscess formation. Thus, end-colostomy diverticulitis should be part of differential diagnosis in presence of these symptoms.

Computed tomography can play a decisive role by determining complication of acute diverticulitis, along with expansion of inflammation and its depth.^[9]^

Acute diverticulitis can be complicated by more severe clinical presentation.^[10]^ In the current case, the fasciitis might have developed as a consequence of the closeness of the inflammation to the muscular fascia. This complication has already been described in acute perforated sigmoid diverticulitis.^[11]^ Surgical management and segmental colonic resection should therefore be considered after acute phase despite lack of robust data in literature.

In conclusion, this case highlighted a particular presentation of acute diverticulitis in a subgroup of population living with end-colostomy. Considering the potential risk of complications associated with this presentation, surgical treatment should be considered after initial antibiotics treatment of acute phase.
